# Hormonal and gene dynamics in de novo shoot meristem formation during adventitious caulogenesis in cotyledons of *Pinus pinea*

**DOI:** 10.1007/s00299-020-02508-0

**Published:** 2020-01-28

**Authors:** José M. Alvarez, Natalia Bueno, Candela Cuesta, Isabel Feito, Ricardo J. Ordás

**Affiliations:** 1 Instituto Universitario de Biotecnología de Asturias, Departamento de Biología de Organismos y Sistemas, Universidad de Oviedo, Oviedo, Spain; 2 Servicio Regional de Investigación Y Desarrollo Agroalimentario de Asturias (SERIDA), Villaviciosa, Spain

**Keywords:** De novo shoot organogenesis, Conifers, Plant growth regulators, Gene expression, Multivariate analyses

## Abstract

**Key message:**

Several members of *WOX* and *KNOX* gene families and several plant growth regulators, basically cytokinins and auxins, play a key role during adventitious caulogenesis in the conifer *Pinus pinea*.

**Abstract:**

Similar to *Arabidopsis thaliana*, *Pinus pinea* shoot organogenesis is a multistep process. However, there are key differences between both species, which may alter the underlying physiological and genetic programs. It is unknown if the genic expression models during angiosperm development may be applicable to conifers. In this work, an analysis of the endogenous content of different plant growth regulators and the expression of genes putatively involved in adventitious caulogenesis in *P. pinea* cotyledons was conducted. A multivariate analysis of both datasets was also realized through partial least squares regression and principal component analysis to obtain an integral vision of the mechanisms involved in caulogenesis in *P. pinea*. Analyses show that cotyledons cultured in the presence of benzyladenine during long times (2–6 days) cluster separately from the rest of the samples, suggesting that the benzyladenine increase observed during the first hours of culture is sufficient to trigger the caulogenic response through the activation of specific developmental programs. In particular, the most relevant factors involved in this process are the cytokinins trans-zeatin, dihydrozeatin, trans-zeatin riboside and isopentenyl adenosine; the auxin indoleacetic acid; and the genes *PpWUS*, *PpWOX5*, *PpKN2*, *PpKN3* and *PipiRR1*. *WUS* is functional in pines and has an important role in caulogenesis. Interestingly, *WOX5* also seems to participate in the process, although its specific role has not been determined.

**Electronic supplementary material:**

The online version of this article (10.1007/s00299-020-02508-0) contains supplementary material, which is available to authorized users.

## Introduction

The formation of adventitious shoots is a complex process that involves the participation of plant growth regulators (PGRs), mainly auxins and cytokinins, with multiple signaling pathways (Kareem et al. 2016; Sang et al. 2018; Tian et al. 2018; Ikeuchi et al. 2019). Three phases have been differentiated during the in vitro caulogenic process: acquisition of morphogenic competence (associated with certain level of cellular dedifferentiation or transdifferentiation); induction of cell identity specification for shoot apical meristem (SAM) formation in response to exogenous PGRs; and shoot development (Christianson and Warnick 1983).

De novo shoot organogenesis has been deeply studied in the model plant Arabidopsis (*Arabidopsis thaliana* L. Heynh). In this organism, in vitro caulogenesis is an indirect process, as it is achieved through a two-step regeneration protocol that involves the culture of root explants in an auxin-rich callus induction medium (CIM) followed by the incubation of explants in a cytokinin-rich shoot induction medium (SIM) (Valvekens et al. 1988; reviewed in Ikeuchi et al. 2019). During CIM incubation, explants progressively acquire competence to respond to the induction stimuli, while within SIM incubation, explants become competent to differentiate into shoots. During these phases, auxin and cytokinin response signals in a mutually exclusive pattern (Sang et al. 2018). In callus masses, the activation of a cytokinin response domain was reported, as type-B ARABIDOPSIS RESPONSE REGULATOR (ARR) transcription factors such as ARR1, ARR10, and ARR12 directly suppress *YUCCA4* (*YUC4*) expression and prevent auxin biosynthesis (Meng et al. 2017). As a result, members of the class III homeodomain-leucine zipper (HD Zip III) transcription factors directly interact with type-B ARRs and induce the expression of *WUSCHEL* (*WUS*) in a selected group of cells, which specifies stem cell fate in the center of the regenerating shoot meristem (Meng et al. 2017; Zhang et al. 2017; Zubo et al. 2017). Afterward, these HD Zip III proteins upregulate other genes that play key roles in the SAM formation and maintenance such as the class I *KNOX* gene *SHOOT MERISTEMLESS* (*STM*) (Shi et al. 2016). Altogether, these processes promote the transition from callus cells into shoot stem cells giving rise to de novo establishment of SAMs.

De novo shoot organogenesis protocols have been also developed in other plant groups such as conifers. In *Pinus* spp*.*, in vitro caulogenesis induction is easily obtained in cotyledons detached from non-germinated mature embryos cultured in the presence of a cytokinin, usually benzyladenine (BA) (Flinn et al. 1988; López et al. 1996). This technique has been used for clonal plant production in some species such as stone pine (*Pinus pinea* L.), a Mediterranean native species that has been economically exploited for its edible seeds or pine nuts (González et al. 1998; Valdés et al. 2001; Moncaleán et al. 2005; Alonso et al. 2006; Cortizo et al. 2009; Cuesta et al. 2009). Apart from its use in breeding programs, *P. pinea* has been proposed as a model for the study of the physiological and molecular basis of caulogenesis in conifers (Cuesta et al. 2009). Unlike Arabidopsis, in vitro caulogenesis in *P. pinea* is an example of direct organogenesis, as cotyledons are competent per se and respond to the induction signal (consisting in the addition of a single PGR to the induction medium), without an intermediate callus formation in a very synchronous fashion (Cuesta et al. 2009). Several studies have shown that the endogenous hormonal content of *P. pinea* cotyledons determined the organogenic capacity (Valdés et al. 2001; Moncaleán et al. 2005; Cortizo et al. 2009; Cuesta et al. 2012). Cotyledons excised from germinated embryos showed a lower organogenic capacity than those excised from non-germinated embryos, which was associated with a reduction in active cytokinins and indoleacetic acid (IAA) endogenous levels (Valdés et al. 2001). Furthermore, the evaluation of the organogenesis response in selected half-sibling families showed that this process is genotype dependent (Cuesta et al. 2008), being connected with the cytokinin content, which significantly differed between families with opposite caulogenesis parameters (Cuesta et al. 2012).

Despite the available information about hormonal content, the knowledge about the underlying molecular mechanisms of de novo shoot formation in conifers, both in vitro and *in planta* is still limited. Previous studies have characterized in *P. pinea* a type-A response regulator (*PipiRR1*) involved in the cytokinin signal transduction pathway and a leucine-rich-repeat protein receptor kinase gene (*PipiCLV1L*) that shows homology with *CLAVATA1* (*CLV1*) gene from Arabidopsis, a member from the central pathway involved in SAM homeostasis maintenance. Even more, both genes are upregulated in the presence of BA during in vitro caulogenesis (Cortizo et al. 2010; Alvarez et al. 2013).

Increasing availability of data on the genome and transcriptome of several gymnosperms facilitates the study of genes involved in SAM formation and maintenance. A recent study in *Pinus pinaster* showed for the first time that conifers contain functional discrete *WUS* and *WUSCHEL-RELATED HOMEOBOX 5* (*WOX5*) orthologs expressed in SAM and root apical meristem (RAM), respectively, suggesting that these genes might play similar roles to those described for their Arabidopsis counterparts (Alvarez et al. 2018). Furthermore, the analysis of *KNOX* gene family in *P. pinaster* (Bueno et al., unpublished work) allowed the identification of four class I *KNOX* genes (*PpKN1*-*PpKN4*), in concordance with several pine and spruce species reports, which showed, based on expression studies and transformation experiments, a similar role in SAM functioning compared to their angiosperm counterparts (Sundås-Larsson et al. 1998; Hjortswang et al. 2002; Guillet-Claude et al. 2004; Belmonte et al. 2007; Larsson et al. 2012).

Our starting work hypothesis is that SAM formation and stem development is a conserved process in the evolution of seed plants and therefore the model proposed for Arabidopsis can be extrapolated to gymnosperms. To contrast this hypothesis, we will need to deepen our knowledge on the physiological and molecular factors involved in the shoot formation in gymnosperms. Plant development is the result of the homeostasis of all endogenous PGRs, with greater or lesser importance of one or the other in each of the processes involved. The experiments performed by Skook and Miller (1957) already showed that cytokinins and auxins play a key role in the induction and development of shoots in tissues grown in vitro. Until recently, most studies focused on these phytohormones as the main modulators of in vitro caulogenesis. However, it is known that all PGRs interact among themselves modifying their responses in a mechanism called cross talk (Skalický et al. 2018). In addition, the preparation of in vitro explants and culture conditions are stressful and, therefore, could modulate hormonal homeostasis. For that reason, in this work we analyzed the endogenous content of several PGRs from different classes of plant hormones, including cytokinins, gibberellins, brassinosteroids, auxins and stress-related PGRs, such as abscisic and salicylic acid, and jasmonates, during the induction phase of de novo shoot formation in *P. pinea*. We also conducted the expression profile analysis of several genes that putatively participate in the signaling pathways involved in SAM formation and maintenance in conifers (several *WOX* and *KNOX* members, *PipiRR1* and *PipiCLV1L*) along this process. In particular, we compared hormonal and gene expression dynamics in BA-treated and non-treated cotyledons to elucidate their involvement in this process. Multivariate analyses were carried out to integrate PGRs dynamics and gene expression analysis during the organogenic process.

## Material and methods

### Explant source and culture conditions

One-year-old mature seeds from open-pollinated *Pinus pinea* trees growing in natural stands were used in this study. Seeds from “ES01 Meseta Norte” provenance were provided by “Servicio de Material Genético del Ministerio de Medio Ambiente” (Spain). After removing the seed coat, megagametophytes were surface sterilized by immersion in 7.5% (v/v) H_2_O_2_ for 45 min, followed by three rinses in sterile double-distilled water, with a final imbibition step in moistened sterile paper for 48 h at 4 °C in darkness.

Cotyledons were then excised from embryos and placed horizontally in 200-mL baby food jars containing 20 mL of Lepoivre medium modified by Aitken-Christie et al. (1988) with half-strength macroelements and supplemented with 3% (w/v) sucrose, 0.8% (w/v) agar (Duchefa, NL) and a final concentration of 44.4 µM BA (Duchefa, NL), adjusting pH to 5.8 before autoclaving (Cuesta et al. 2009). Cotyledons cultured in the same medium without BA were used as control. Cultures were maintained in a growth chamber at 25 ± 2 °C with a 16-h photoperiod at a photon flux of 20 ± 5 µmol m^−2^ s^−1^.

Batches of about 30 cotyledons cultured in the presence and absence of BA (hereafter BA-treated and control cotyledons, respectively) were harvested after different times of culture (0, 0.25, 0.5, 1, 2, 4 and 6 days), rinsed with distilled water, drained with filter paper, immediately frozen in liquid nitrogen and stored at − 80 °C until analysis was carried out. Experiments were repeated three times.

### Quantification of plant growth regulators (PGRs) during adventitious caulogenesis in *Pinus pinea*

#### PGR extraction

The extraction of multiple PGRs (abscisic acid, ABA; indoleacetic acid, IAA; benzyladenine, BA; castasterone, BK; dihydrozeatin, DHZ; dihydrozeatin riboside, DHZR; gibberellin GA_4_; isopentenyl adenine, iP; isopentenyl adenosine, iPA; jasmonic acid, JA; salicylic acid, SA; trans-zeatin, tZ; and trans-zeatin riboside, tZR) was performed following the protocol described by Delatorre et al. (2017). Samples from pooled cotyledons (100 mg of fresh weight were homogenized in liquid N_2_ in a bead mill homogenizer Silamat S6 (Ivoclar Vivadent, Spain) and re-suspended in 1 mL of extraction buffer (2-propanol/H_2_O/HCl 37% 2:1:0.002, v/v/v) in 2-mL Safe Lock propylene tubes (Eppendorf Ltd, Germany). Internal standards d_7_-BA, d_3_-DHZ (3.75 ng); d_6_-SA (7.5 ng); d_6_-ABA, d_5_-IAA, d_2_-GA_9_ (15 ng); and d_5_-BK (30 ng) were added at this point to assess recovery rates. All of them were supplied by Olchemim Ltd. (Czech Republic) except d_6_-SA (Sigma Aldrich, St. Louis, MO, USA).

After agitating by repeated inversion (60 rpm) for 30 min at 4 °C in the dark, the resulting suspension was transferred to Teflon tubes (Oak Rifge Centrifuge Tube, Thermo Scientific, England) and 1.8 mL of dichloromethane was added, agitating for an additional 30 min at the same conditions. The organic lower layer was collected and a re-extraction of the upper phase was carried out. The organic phase was then concentrated in 2-mL glass vials under N_2_ flow and stored at − 20 °C until analysis.

#### Quantification by QQQ-MS/MS

The resulting dried extracts were resuspended in 150 μL of 100% methanol by vortexing (1 min) and sonication (5 min) and filtered through a 0.2 μm regenerated cellulose Captiva Premium Syringe filter (Agilent Technologies, California, USA). All compounds were separated and quantified by ultrahigh performance liquid chromatography coupled with tandem mass spectrometry detectors (UHPLC-MS/MS) in a 6460 Triple Quad LC/MS (Agilent Technologies) following the protocol described by Delatorre et al. (2017). A chromatographic separation was made using a reverse phase column (Zorbax SB-C18 2.1 × 50 mm column). The column was held at 40 °C and the mobile phase used in the chromatography consisted of (A) 99.9% MeOH: 0.1% COOH and (B) ammonium formate (10 mM, pH 4). A linear gradient of MeOH from 10 to 50% and then reaching 100% in 7 and 2 min, respectively, was used for compound elution. PGRs were quantified by dynamic multireaction monitoring (MRM) of their [M + H] + and the appropriate product ions, using optimized cone voltages and collision energies for diagnosis of each PGR analyzed.

### PGR immunolocalization during adventitious caulogenesis in *Pinus pinea*

#### Cotyledon fixation and sectioning

Fixation was performed as described by De Diego et al. (2013). Cotyledons cultured in the presence and absence of 44.4 µM BA during 0, 0.5, 2 and 6 days were immediately fixed for 24 h at 4 °C under vacuum in a fixation solution consisting of 4% (w/v) paraformaldehyde, 4% (w/v) 1-ethyl-3-(3-dimethyl-aminopropyl) carbodiimide (EDAC; Sigma-Aldrich Co., St Louis, MO, USA), and 0.1% (v/v) Triton X-100 (Sigma-Aldrich). Samples were then washed three times for 10 min each in phosphate-buffered saline (PBS) and stored in PBS containing 0.1% (w/v) paraformaldehyde at 4 °C until use.

Cotyledons were immersed in Tissue-Tek (Sakura Finetek USA, Inc., Torrance, CA, USA) and frozen at − 20 °C. Longitudinal sections of 30 µm were obtained in a cryomicrotome CH1510-1 (Leica Microsystems GmbH Wetzlar, Germany) and mounted on Menzel-Gläser Superfrost Ultra Plus slides (Thermo-Scientific, Waltham, Massachusetts, USA), which were conserved at − 20 °C until analysis.

#### Immunolocalization using benzyl adenosine (BAR), DHZR and iPA polyclonal antibodies

Immunolocalization of benzyl adenosine (BAR), DHZR and iPA was performed according to the method described by De Diego et al. (2013). About five cotyledons detached from different embryos were used for each treatment. Sections of cotyledon tips were washed in PBS for 1 min, dehydrated in an ascendant ethanol series (25%, 50%, 75% and 100%, v/v) for 5 min each, and then rehydrated in a descendant ethanol series (100%, 75%, 50% and 25%, v/v) for 5 min each. Finally, sections were washed in PBS containing 0.1% (v/v) Tween 20 for 30 min, and then in PBS for 5 min.

Sections were treated with blocking solution (5% bovine serum albumin (BSA) in PBS, w/v) to reduce nonspecific binding for 30 min. Then, samples were incubated with BAR, DHZR and iPA polyclonal primary antibodies (Agrisera, Vännäs, Sweden) overnight at 4 °C. Dilutions 1/50 in 1% BSA/PBS were used for BAR and DHZR primary antibodies, and dilution 1/100 in 1% BSA/PBS was used for iPA primary antibody. After washing twice with 0.1% (v/v) Tween 20 in PBS for 10 min, sections were incubated with Alexa Fluor 488 goat anti-rabbit secondary antibody (Life technologies, Carlsbad, California, USA) diluted 1/25 in 1% BSA/PBS for 1 h in darkness. Sections with only secondary antibody and without antibodies were also included as negative controls to assess nonspecific binding and tissue autofluorescence, respectively. Two wash steps with 0.1% (v/v) Tween 20 in PBS for 10 min were carried out to reduce nonspecific binding. Sections were then incubated with 4,6-diamidino-2-phenylindole (DAPI; AppliChem GmbH, Darmstadt, Germany) 1 μg mL^−1^ for 15 min. Finally, sections were washed three times with autoclaved ddH_2_O for 5 min, air-dried and mounted with Mowiol® 4–88 (AppliChem GmbH).

Sections were visualized using a Leica DM2000 fluorescence microscope (Leica Microsystems, Wetzlar, Germany). Fluorescence from the apical portion of three to five cotyledons per treatment was quantified by ImageJ software (https://fiji.sc/) following the procedure described by Burgess et al. (2010).

### Expression analysis of genes putatively involved in shoot apical meristem formation and maintenance during caulogenic induction in *Pinus pinea* by quantitative real-time PCR (RT-qPCR)

#### RNA isolation and cDNA synthesis

RNA from explants was extracted using the NucleoSpin RNA Plant kit (Macherey–Nagel, Germany) according to the manufacturer’s instructions. A microgram of total RNA was reverse transcribed with the High-Capacity cDNA Reverse Transcription Kit (Applied Biosystems Inc., Foster City, CA, USA) following the manufacturer’s protocol.

#### RT-qPCR

Based on the role in SAM formation and maintenance, the expression pattern of three *WOX* genes (*PpWOX5*, *PpWOXX* and *PpWUS*; Acc. ALN42234, ANC94876 and ALN42231, respectively), four class I *KNOX* genes (*PpKN1-KN4*; Acc. KT356208, KT356209, KT356211 and KT356210, respectively), *PipiRR1* (Acc. FJ717710) and *PipiCLV1L* (Acc. HQ377525) was analyzed during in vitro caulogenic induction by RT-qPCR. Two ESTs (Pp4C3 and Pp5F10; Acc. EC611869 and EC428628, respectively) were used as endogenous reference genes, as had been shown to be constitutively expressed during this process (Cortizo et al. 2010). RT-qPCR was performed in an ABI PRISM 7900HT instrument (Applied Biosystems). Individual reactions were assembled in triplicate with oligonucleotide primers (0.20 µM each), 5 μL of Fast SYBR Green Master Mix (Applied) plus 100 ng of cDNA in a final volume of 10 μL. Primers for each gene were designed with Primer3 software (Rozen and Skaletsky 2000) to amplify a fragment between 60 and 150 bp of the target cDNA with a 60 °C Tm and a G + C content of 40–60% (Udvardi et al. 2008) (Online Resource 1). Amplifications were performed using the following standard protocol: 95 °C 20 s, 45 cycles of 95 °C 1 s and 60 °C 20 s, with a final melting curve analysis to dismiss the presence of non-specific products. Negative controls (no template) and RT-controls (non-retrotranscribed RNA) were also included to assess amplification product specificity.

Data analysis was performed according to the comparative Ct method (Livak and Schmittgen 2001) through LinRegPCR software, incorporating the mean PCR efficiencies of the target and endogenous genes in the plate. Relative abundance of each transcript was calculated as the mean of three technical replicates and normalized to the mean expression value of the reference genes in each sample.

### Data analysis

A randomized experimental design was applied (Compton 1995) to study the quantitative variables. Three independent experiments were carried out, with three samples per time point that were taken from different culture vessels. The statistical analysis was performed by the Kruskal–Wallis test for independent samples using the SPSS Statistics software (Chicago, IL, USA).

In addition, multivariate analyses of the endogenous hormonal content and gene expression data were performed. PGRs content and gene expression data were analyzed by principal component analysis (PCA), and a partial least squares (PLS) regression was performed for the integration of hormonal content and gene expression data to find the relationships between both datasets. For convenience, data were grouped into two categories for each treatment: short culture times, including samples from 0 to 1 day (BA_ST_ and C_ST_), and long culture times, including samples from 2 to 6 days (BA_LT_ and C_LT_). Both PCA and PLS analyses were performed through R software v3.5.1 (https://www.r-project.org/).

## Results

### Analysis of the endogenous content and distribution of different plant growth regulators (PGRs) during caulogenic induction in *Pinus pinea*

**Fig. 1:**
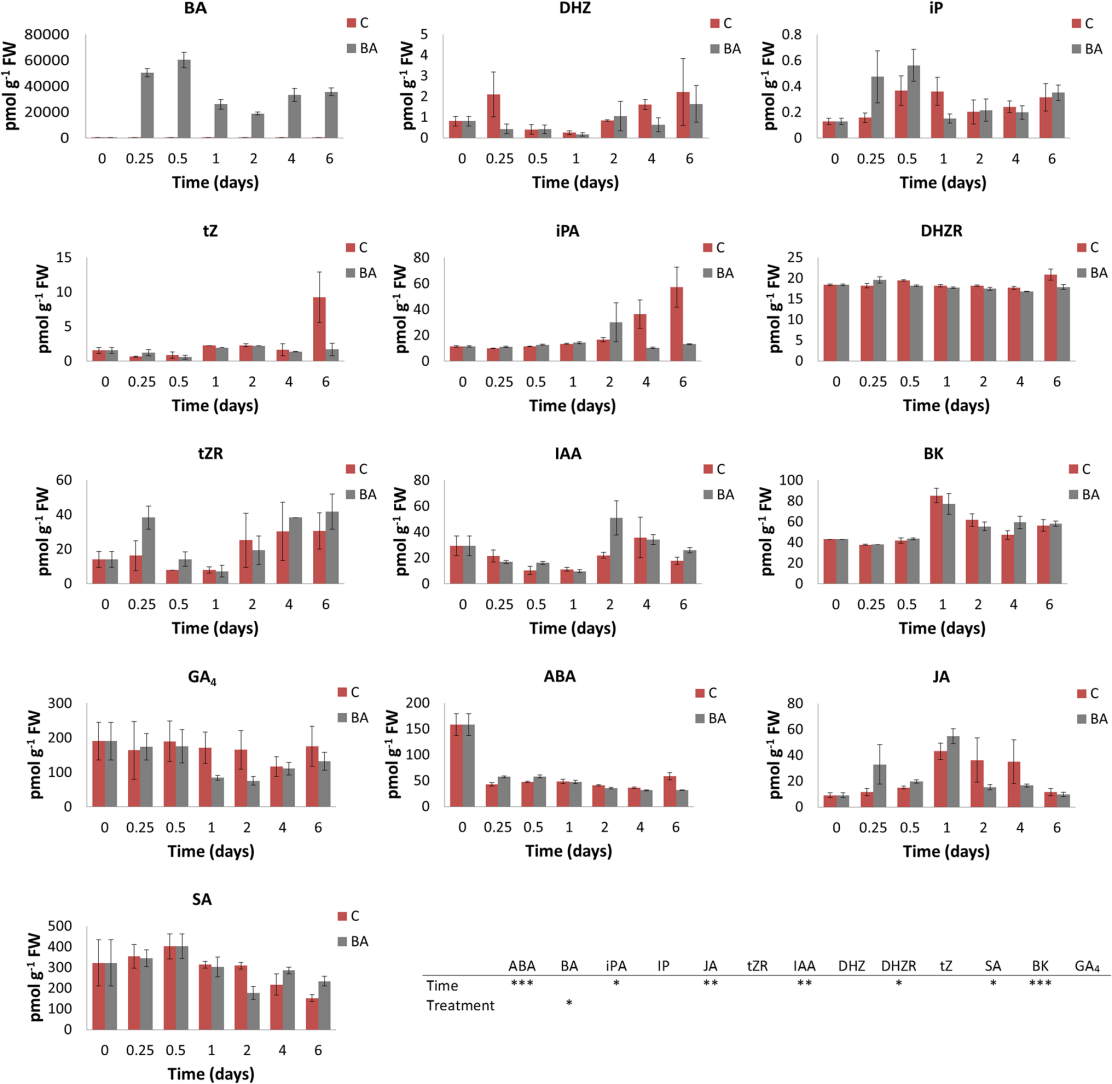
Content profile of the main plant growth regulators (PGRs), extracted from *Pinus pinea* cotyledons cultured in the presence of benzyladenine (BA-treated cotyledons, BA) and absence of exogenous hormone (control cotyledons, C): *BA* benzyladenine, *DHZ* dihydrozeatin, *DHZR* dihydrozeatin riboside, *tZ* trans-zeatin, *tZR* trans-zeatin riboside, *iP* isopentenyl adenine, *iPA* isopentenyl adenosine, *IAA* indoleacetic acid, *GA*_*4*_BK castasterone, gibberellin A4, abscisic acid, ABA; jasmonic acid, JA; salicylic acid, SA. Results are expressed as mean values ± standard error. Quantitative data were analyzed by Kruskal–Wallis test. Asterisks indicate significant differences between treatments or times of culture (*P ≤ 0.05; **P ≤ 0.01;***P ≤ 0.001

The overall dynamics of PGR content in BA-treated and control cotyledons over different culture times are shown in Fig. 1. BA endogenous levels presented the biggest differences between explants cultivated in the presence and absence of BA. It showed a significant increase in BA-treated cotyledons, with maximum levels at 12 h, and then decreased until 2 days, with a non-significant slight increase afterward. The free base cytokinins DHZ and iP showed non-significant similar dynamics in BA-treated and control cotyledons, while tZ and iPA presented a higher concentration in control cotyledons at 6 days. Regarding other ribosylated forms, DHZR and tZR content was stable during caulogenic induction in both types of explant.

Regarding auxins, it is remarkable that, at 2 days of induction, the IAA content increased in BA-treated cotyledons with respect to control cotyledons. No significant differences between control and BA-treated cotyledons were found for the brassinosteroid BK along the process. In the case of GA_4_, its levels were unaltered in control cotyledons along the process, whereas they were reduced in the presence of BA from 0.5 to 1 days.

Three PGRs involved in plant stress responses were analyzed in this study: ABA, JA and SA. General trends were very similar under both BA-treated and control cotyledons. In detail, a significant reduction of ABA levels was observed after 0.25 days in both treatments, being maintained at similar levels throughout the whole process. JA reached a peak at 1 day in both treatments, and then showed a significant decrease in case of BA-treated cotyledons, with a progressive reduction in control cotyledons. Concerning SA levels, a reduction was observed from 0.5 days on in both control and BA-treated cotyledons, which was more pronounced in the latter case. Whereas SA levels kept reducing progressively throughout the experiment in control cotyledons, a significant rise was observed in BA-treated cotyledons from 4 days on.

**Fig. 2:**
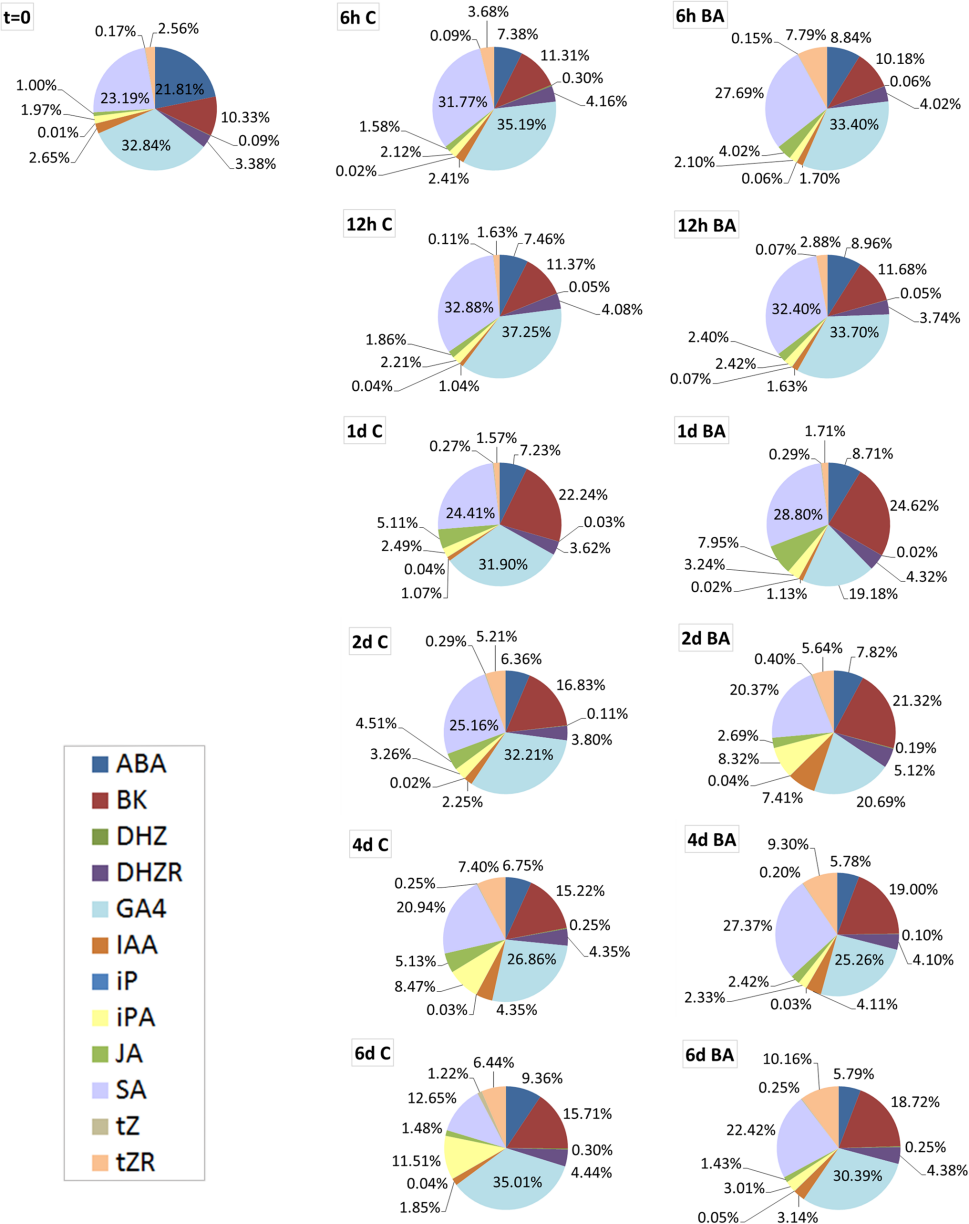
Proportion of different plant growth regulators (PGRs) during de novo shoot formation in *Pinus pinea* expressed in percentage of each hormone with respect to the total of tested compounds and excluding benzyladenine (BA) (color figure online)

To study the hormonal profile that defines each culture stage, the relative content of each hormone was calculated. As expected, BA showed the highest proportion in BA-treated cotyledons, reached a maximum at 0.5 days (98.75%) and maintained more than 97% throughout the experiment, whereas in the control cotyledon case, BA only represented 6% (0.25 days) to 20% (1 days) (data not shown). To compare both types of explants, the percentage of each PGR excluding BA was obtained (Fig. 2). In this case, GA_4_ was the most abundant PGR, followed by SA and BK. Regarding cytokinins, in the beginning ribosides were more abundant than free bases, keeping this dynamic along the process in both control and BA-treated cotyledons. In fact, iP, DHZ and tZ constituted the lowest content from all PGRs studied.

### PGR immunolocalization during adventitious caulogenesis in *Pinus pinea*

**Fig. 3:**
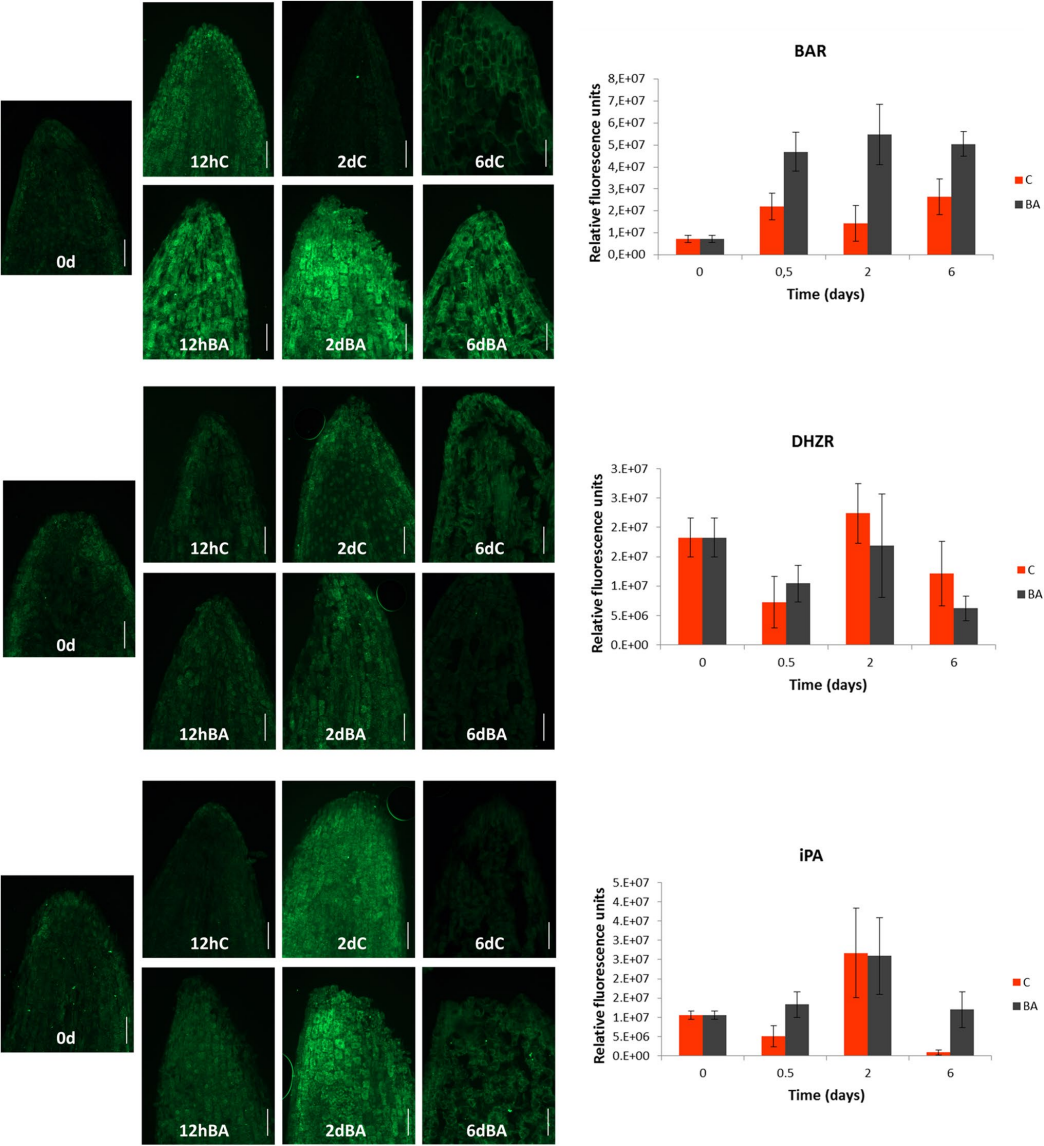
Immunodetection of benzyl adenosine (BAR), dihydrozeatin riboside (DHZR) and isopentenyl adenosine (iPA) in longitudinal sections of *Pinus pinea* cotyledons cultured in the presence and absence of benzyladenine (BA) during 0 day, 12 h, 2 days and 6 days. Alexa 488 secondary antibody (green signal) was used. Fluorescence quantification was performed following the procedure described by Burgess et al. (2010) through ImageJ software (https://fiji.sc/). BA-treated cotyledons (BA): black bars; non-treated cotyledons (C): red bars. Results are expressed as the mean of the fluorescence values ± standard error from 3–5 cotyledons per treatment. Bar, 100 µm

Immunolocalization of BAR, DHZR and iPA, performed in the tip of cotyledons, supported the data obtained through UHPLC-MS/MS to a great extent, with some differences probably due to the area of analysis, as it was only focused on the apical part, instead of the whole cotyledon (Fig. 3).

In general terms, BAR showed higher levels in BA-treated cotyledons, with a maximum peak at 2 days, meanwhile DHZR showed variability in its response, with no clear differences between treatments. In regard to iPA, samples showed a rise along the first period (0–2 days), with a decrease observed at 6 days, especially pronounced in the control ones.

### Expression analysis of genes putatively involved in shoot apical meristem formation and maintenance during caulogenic induction in *Pinus pinea* by quantitative real-time PCR (RT-qPCR)

**Fig. 4:**
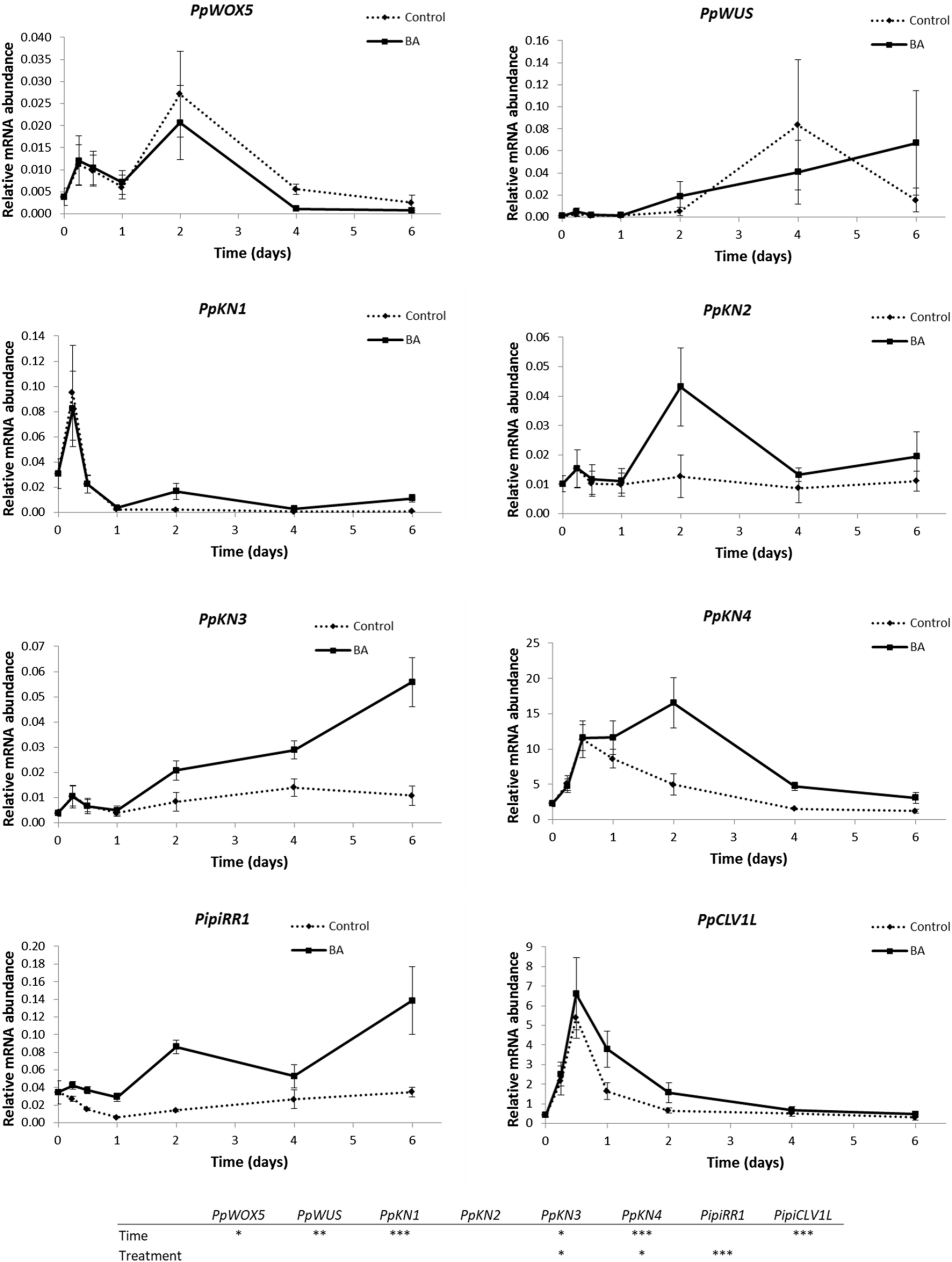
Quantitative real-time (RT-qPCR) analysis of the mRNA abundance of several members from *WOX* (*PpWOX5*, *PpWUS*) and *KNOX* (*PpKN1*, *PpKN2*, *PpKN3*, *PpKN4*) gene families, *RESPONSE REGULATOR1* (*PipiRR1*) and *CLAVATA1-LIKE* (*PipiCLV1L*) in *Pinus pinea* cotyledons cultured in the presence and absence of benzyladenine (BA) during the induction phase of adventitious caulogenesis. Results are expressed as mean values of the relative expression ± standard error. Quantitative data were analyzed by Kruskal–Wallis test. Asterisks indicate significant differences between treatments or times of culture (*P ≤ 0.05; **P ≤ 0.01; ***P ≤ 0.001

Results of the expression analysis of three genes from the *WOX* gene family (*PpWUS, PpWOXX* and *PpWOX5*), the four class I *KNOX* genes (*PpKN1*-*PpKN4*), *PipiRR1*, and *PipiCLV1L* are shown in Fig. 4.

The mRNA level of *PpWUS* increased progressively and significantly during the organogenic process in BA-treated cotyledons, showing an erratic pattern in control cotyledons. Levels of *PpWOX5* transcripts increased during the first hours of culture in control and BA-treated cotyledons, with a later decrease in both types of explant. In regard to *PpWOXX* expression, it was below the detection limit for most samples (data not shown).

Overall, *PpKN* genes seemed to be upregulated in the presence of BA. *PpKN1* levels underwent an important increase at 0.25 days, with a later dramatic drop in both types of explants. Regarding *PpKN2*, whereas its levels were maintained quite constant in control cotyledons, its expression increased at 2 days of culture in BA-treated cotyledons, followed by a slight decrease and then remained upregulated in the presence of BA. *PpKN3* expression levels were very similar in BA-treated and control cotyledons during the first hours of culture, and increased progressively and significantly from 1–2 days on in BA-treated cotyledons. For its part, *PpKN4* expression reached a significant peak at 2 days of culture in BA-treated cotyledons. Then, its levels decreased and remained about two to three times upregulated in the presence of the hormone.

The gene *PipiRR1* was significantly upregulated in the presence of BA along the process. Finally, in the case of *PipiCLV1L* expression, it showed a significant increase during the first hours of culture in control and BA-treated cotyledons, with a later decrease for both types of material, being more pronounced in control cotyledons.

### Multivariate analyses of endogenous hormonal content and gene expression data

**Fig. 5:**
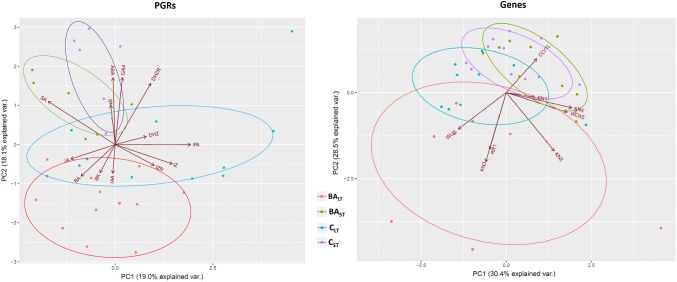
Analysis of PGRs content and gene expression data by principal component analysis (PCA). BA_ST_: short times (0–1 days), BA-treated cotyledons (green); BA_LT_: long times (2–6 days), BA-treated cotyledons (red); C_ST_: short times (0–1 days), control cotyledons (purple); *C*_LT_: long times (2–6 days), control cotyledons (blue) (color figure online)

The PCA analyses of PGRs content and gene expression data revealed clear differences between short (0–1 days) and long (2–6 days) times of culture (Fig. 5). Whereas no differences were found between control and BA-treated cotyledons at short times, both treatments clustered separately for long times. These differences were mainly explained by BA, BK and IAA for PGR content PCA, and *PpWUS*, *PpKN2*, *PpKN3* and *PipiRR1* for gene expression PCA.

**Fig. 6:**
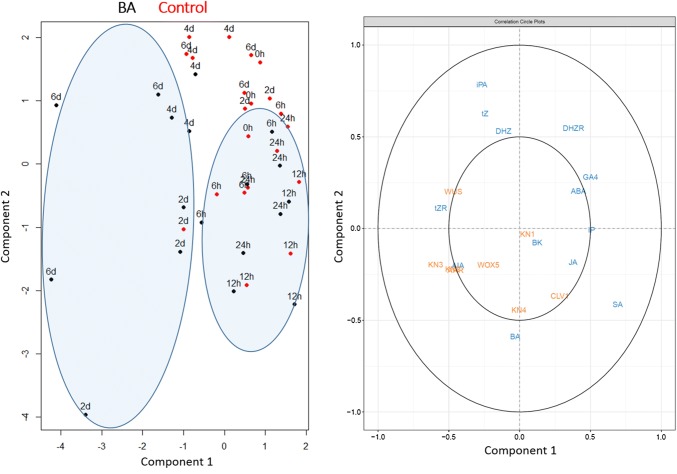
Correlation loading plot obtained by partial least squares (PLS) regression using plant growth regulator content as the *X* matrix and gene expression data as the *Y* matrix

PLS regression analysis of PGR content and gene expression data showed that samples cultured in the presence of BA during long times (2–6 days) clustered separately from the rest of the samples. This distribution was mainly explained by the PGRs tZ, DHZ, iPA, tZR and IAA, and the genes *PpWUS*, *PpWOX5*, *PpKN2*, *PpKN3* and *PipiRR1* (Fig. 6). It is also remarkable that samples cultured in the presence and absence of BA during short times (0–1 days) grouped together, and stress-related PGRs (ABA, JA and SA), GA_4_ and iP were mainly responsible for this distribution.

## Discussion

In vitro caulogenesis in *P. pinea*, which consists of the development of adventitious buds on the surface of cotyledons cultured in a BA-containing medium, is a method used for the micropropagation of selected genotypes on a large scale (Alonso et al. 2006), but it also constitutes an ideal model for the study of the underlying processes of the SAM formation and maintenance in conifers (Valdés et al. 2001; Moncaleán et al. 2005). Similar to Arabidopsis, *P. pinea* shoot organogenesis in tissue culture is a multistep process (Moncaleán et al. 2005; Cuesta et al. 2009). However, there are key differences between both species, which may alter the underlying physiological and genetic programs. Whereas shoot regeneration in Arabidopsis is an indirect process, it has been reported that *P. pinea* cotyledons are competent per se to respond to organogenic signals and become committed to shoot regeneration (Cortizo et al. 2009; Cuesta et al. 2009). Therefore, it is unknown if the models of genic expression during angiosperm development may be applicable to conifers.

Previous works support the hypothesis that differences in hormonal contents may induce shoot organogenesis (Cuesta et al. 2012). The present data will help to understand the behavior of gene expression profiles related to caulogenic induction, connecting their response magnitude with the PGRs levels.

The minimum induction period for *P. pinea* cotyledons cultured in a medium containing 44.4 µM BA is 0.25 days and the maximum response is achieved after 2–4 days of BA exposure (Moncaleán et al. 2005; Cuesta et al. 2009). In these conditions, promeristemoids, later developing into meristemoids and shoot primordia, are present at 0.5 days in BA-treated cotyledons, and its appearance is preceded by intense mitotic activity (Cuesta et al. 2009). This indicates that shoot commitment or induction, in which the fate of competent cells is specified for the formation of shoot buds, takes place in the first hours of culture in the presence of BA. In addition, the application of this PGR would only be necessary in the first hours of culture to induce caulogenesis (Cortizo et al. 2009; Cuesta et al. 2009). Consistently with these results, a huge increase of the endogenous BA levels in BA-treated cotyledons was observed during the first hours of culture, which is due to the high absorption rate of exogenous BA (Cuesta et al. 2009). Similar to Cuesta et al. (2009) observations, three phases could be distinguished during caulogenic induction based on BA dynamics: (i) from 0 to 0.5 days, characterized by a high BA uptake rate, reaching the upper peak of BA content, which is fitting with the presence of the first promeristemoid structures; (ii) from 0.5 to 2 days, characterized by a pronounced decrease in BA concentration; and (iii) from 2 to 6 days, when BA levels slightly increased again.

Despite the relevance of the BA increase during the first hours of culture in BA-treated cotyledons, multivariate analyses (PLS regression and PCA) showed that the main differences for most PGRs and genes analyzed between control and BA-treated cotyledons were found at longer culture times (from 2 days on), suggesting that the initial BA peak triggers the caulogenic response through later changes in other PGRs and gene expression levels. In particular, BA-treated cotyledons cultivated during long times in the induction medium constituted a clearly separated cluster. Based on PLS regression results, this distribution was mainly explained by the cytokinins iPA, tZ, tZR and DHZR, the auxin IAA, and the genes *PpWUS*, *PpWOX5*, *PpKN2*, *PpKN3* and *PipiRR1*.

The ribosides iPA and tZR and the free bases tZ and DHZ might perform a relevant role in the caulogenic process. iPA is considered the precursor in the isoprenoid cytokinin biosynthetic pathway and it was previously reported to replace BA as inducer of the morphogenic responses (Charrière et al. 1999; Cuesta et al. 2012). Free bases are highly active forms, especially tZ, and it has been proposed that they are rapidly used during caulogenic induction, preventing their accumulation at high levels (Moncaleán et al. 2005; Stirk et al. 2005; Cuesta et al. 2012).

The participation of IAA in caulogenic induction in *P. pinea* could be related to the response of the explant to exogenous BA application to reach an optimum cytokinin:auxin ratio for de novo meristem formation. It was established that endogenous auxins together with cytokinins play a key role in initiation and proliferation of meristems, as increasing or decreasing the ratio of cytokinin:auxin in the culture medium induced the regeneration of shoots or roots, respectively (Skoog and Miller 1957). In Arabidopsis, it has been reported that genes involved in auxin biosynthesis are induced during the incubation in the cytokinin-rich medium (Cheng et al. 2013) in a non-overlapping region with the cytokinin response domain, although the role of auxins during shoot regeneration is not completely established (Sang et al. 2018; Ikeuchi et al. 2019). Our results showed that the level of IAA increased in BA-treated cotyledons after active free cytokinins such as BA, in agreement with other authors who found a similar increase of endogenous IAA levels in explants during adventitious organogenesis in other species (Mercier et al. 2003; Malá et al. 2006).

Regarding PGRs involved in stress tolerance, no dramatic changes were observed in their content between BA-treated and non-treated cotyledons along the organogenic process. This is in concordance with Pérez-Jiménez et al. (2014), who did not find any significant differences in JA content in *Prunus persica* cultures with different organogenic capacity. Interestingly, these PGRs could be responsible for grouping together cotyledons cultured in the presence and absence of BA at short times according to the multivariate analysis results. This could be due to the fact that cotyledon excision from mature embryos and transference to an artificial culture media cause a stress that affects their hormonal profile. On the other hand, it seems that the application of a very high exogenous BA concentration in the medium with a high rate of absorption did not trigger specific stress tolerance responses in BA-treated explants. Overall, it seems that stress-related PGRs do not have a main role in caulogenesis in this species. Similarly, GA_4_ and iP levels were slightly affected by BA treatment along the process, but both PGRs contributed to the distribution mentioned above. The brassinosteroid BK could play some role at long times according PCA. However, this role could not be exclusive for BA-treated cotyledons, since no significant differences in BK content were found between control and BA-treated cotyledons at long times.

Some members from *WOX* and *KNOX* gene families seem to play a key role during caulogenesis in *P. pinea*, similar to Arabidopsis (Ikeuchi et al. 2019). At short culture times, no big differences between control and BA-treated cotyledons were found for most of them, since only a small number of cells within the explant respond to the induction signal (Thorpe 1980; Alonso et al. 2007). As expected, the peak of BA preceded the increase in the expression of several of the genes analyzed in this work. PLS regression and PCA analyses suggest a pivotal role for *PpWUS*, *PpWOX5*, *PpKN2*, *PpKN3* and *PipiRR1* in the caulogenic process. In Arabidopsis, *WOX5* expression is induced during acquisition of competence in the auxin-rich medium (Sugimoto et al. 2010), but caulogenesis in *P. pinea* is a direct process without an intermediate callus phase. Although it has been proposed that *PpWOX5* participates in RAM organization in conifers, expression of this gene was detected in other tissues apart from root tips such as shoot apexes, hypocotyls and cotyledons (Alvarez et al. 2018). Therefore, *WOX5* could perform additional roles to those traditionally described in angiosperms.

Several class I *KNOX* genes were shown to have an important role in caulogenic induction. Analysis of *STM* expression during adventitious caulogenesis in Arabidopsis revealed that it was expressed at low levels during CIM incubation and also upregulated during SIM incubation at the time of shoot commitment (Cary et al. 2002). Based on PCA and PLS regression analyses, the main differences between control and BA-treated cotyledons for long times of culture were caused by *PpKN2* and *PpKN3* genes. Class I *KNOX* gene expression was analyzed during somatic embryogenesis in *Picea abies* to determine their specific roles during SAM formation (Larsson et al. 2012): *KN3* ortholog was expressed in somatic embryogenic lines able to develop fully mature cotyledonary embryos and inhibited in lines with altered SAM development, suggesting that this gene is essential for SAM formation. Conversely*,* the *P. abies KN2* ortholog was shown to be upregulated before SAM formation during somatic embryogenesis both in competent and non-competent embryogenic lines, indicating that this gene has a wider general role during embryogenesis. Our results showed that *PpKN2* reached a peak in BA-treated cotyledons at 2 days of culture, coinciding in the time when cells become determined to develop and differentiate into adventitious shoots (Cuesta et al. 2009). This could suggest an important role in caulogenic determination. Regarding *PpKN3*, its increased levels in the presence of BA after the caulogenic determination might indicate that this gene is involved in meristem organization. Type-A response regulators are upregulated during de novo shoot formation in Arabidopsis (Che et al. 2002), acting as negative regulators of cytokinin signaling and its expression is rapidly induced by this PGR (D’Agostino et al. 2000). The role of *PipiRR1* in adventitious caulogenesis in *P. pinea* was previously reported by Cortizo et al. (2010), and confirmed in the present report.

In conclusion, the results of this work demonstrate that cytokinins play an active role during the in vitro induction and formation of adventitious shoot meristemoids in cotyledons of *P. pinea*, suggested by the initial peak of BA triggering the response through subsequent changes in other PGRs and levels of gene expression. The most relevant factors involved in this process are cytokinins tZ, DHZ, tZR and iPA; the auxin IAA; and the genes *PpWUS*, *PpWOX5*, *PpKN2*, *PpKN3* and *PipiRR1*. *PpWUS* is functional in pines and has an important role in caulogenesis. Interestingly, *PpWOX5* also seems to participate in the process, although its specific function has not been determined. The output of the present work revealed the complexity of in vitro caulogenesis in a relatively simple tissue culture system, which provide a useful base for following further investigation of the function and interaction of different PGRs and genes, specifically those from the *WOX* and *KNOX* families, elucidating if the model of genic expression during organogenic processes in angiosperm development may be valid in conifers.

## Electronic supplementary material

Below is the link to the electronic supplementary material.

Supplementary file1 (DOCX 17 kb)
