# Supplementary material for: Hormonal and gene dynamics in de novo shoot meristem formation during adventitious caulogenesis in cotyledons of Pinus pinea
Source: Plant Cell Rep. 2020 Jan 28;39(4):527–41. doi: 10.1007/s00299-020-02508-0 (PMC7067738; doi:10.1007/s00299-020-02508-0)
Supplement: Supplementary file 1 — Supplementary file1 (DOCX 17 kb) [file 299_2020_2508_MOESM1_ESM.docx]

**Title:** Hormonal and gene dynamics in *de novo* shoot meristem formation during adventitious caulogenesis in cotyledons of *Pinus pinea*

**Journal name:** Plant Cell Reports

**Authors:** José M Alvarez*^1^, Natalia Bueno*^1^, Candela Cuesta^1^, Isabel Feito^2^, Ricardo J Ordás^$1^

**Address:**

^1^ Departamento de Biología de Organismos y Sistemas, Universidad de Oviedo, Spain

^2^ Servicio Regional de Investigación y Desarrollo Agroalimentario de Asturias (SERIDA), Spain

* José M Alvarez and Natalia Bueno contributed equally to this work.

^$^ Corresponding author:

Telephone: +34 985 45 81 80

Email: [rordas@uniovi.es](mailto:rordas@uniovi.es)

**Online Resource 1**. Primers used for gene expression analysis during adventitious caulogenesis in *Pinus pinea* by quantitative real time PCR (RT-qPCR).

| Gene/EST | Name | Sequence (5’ → 3’) |
| --- | --- | --- |
| *PipiCLV1L* | **PipiCLV1L-F** | ATGGCCAAATTCCTGTTCAA |
|  | **PipiCLV1L-R** | TCCAATGCCAGAGGGAATAG |
| *PipiRR1* | **PipiRR1-F** | CAGAAGGCGCTCAAGAGTTT |
|  | **PipiRR1-R** | TTGTTGGTCCCTGGATCTTC |
| *Pp4C3* | **Pp4C3-F** | TTCTCCCAGTTCTCCCAATG |
|  | **Pp4C3-R** | ATTGCAACCGGAAACAGAAC |
| *Pp5F10* | **Pp5F10-F** | CCCTGTTAGACGAAGGGTCA |
|  | **Pp5F10-R** | CTGGCCGAAAGCCATAAATA |
| *PpKN1* | **PpKN1-RealTime-F** | CGAACACCAAGACCCACAAC |
|  | **PpKN1-RealTime-R** | AGTCAATATCTCGCAGTAAGCCT |
| *PpKN2* | **PpKN2-RealTime-F** | GCACAATGACGACGAGTATGCCT |
|  | **PpKN2-RealTime-R** | CCCGTGCTGCCGTTTTAGCTT |
| *PpKN3* | **PpKN3-RealTime-F** | CTGAGCAGGCCGTCATCTCT |
|  | **PpKN3-RealTime-R** | CTCCCCTCTTCTTTCCTGCGG |
| *PpKN4* | **PpKN4-RealTime-F** | CGCAGGTCAGGTTGAAGGTC |
|  | **PpKN4-RealTime-R** | GTTCTTGTGGTGGGAAATGACCA |
| *PpWOX5* | **PpWOX5-RealTime-F** | TGGCACATCATATGCTCCAGCTC |
|  | **PpWOX5-RealTime-R** | TGCAGCTCTCATTTTCTGCGCT |
| *PpWOXX* | **PpWOXX-RealTime-F** | CGCAGCATTCAGGAAGCTCATGC |
|  | **PpWOXX-RealTime-R** | CACCGACGGTCATCTCGGACG |
| *PpWUS* | **PpWUS-RealTime-F** | AGCGCCATGAACGTGGCTG |
|  | **PpWUS-RealTime-R** | GCTTCACAGCGTACATTTGCTCC |
